# Investigating Charge-Induced Transformations of Metal Nanoparticles in a Radically-Inert Liquid: A Liquid-Cell TEM Study

**DOI:** 10.3390/nano14211709

**Published:** 2024-10-26

**Authors:** Kunmo Koo, Jong Hyeok Seo, Joohyun Lee, Sooheyong Lee, Ji-Hwan Kwon

**Affiliations:** 1 Korea Research Institute of Standards and Science, Daejeon 34113, Republic of Korea; kunmo.koo1@gmail.com (K.K.); j.h.seo@kriss.re.kr (J.H.S.); joohyun.lee@kriss.re.kr (J.L.); 2 Department of Nano Convergence Measurement, Korea University of Science and Technology, Daejeon 34113, Republic of Korea

**Keywords:** liquid-phase transmission electron microscopy, Au nanoparticle, phase transition

## Abstract

We present a novel in situ liquid-cell transmission electron microscopy (TEM) approach to study the behavior of metal nanoparticles under high-energy electron irradiation. By utilizing a radically-inert liquid environment, we aim to minimize radiolysis effects and explore the influence of charge-induced transformations. We observed complex dynamics in nanoparticle behavior, including morphological changes and transitions between amorphous and crystalline states. These transformations are attributed to the delicate interplay between charge accumulation on the nanoparticles and enhanced radiolysis, suggesting a significant role for charge-assisted processes in nanoparticle evolution. Our findings provide valuable insights into the fundamental mechanisms driving nanoparticle behavior at the nanoscale and demonstrate the potential of liquid-cell TEM for studying complex physicochemical processes in controlled environments.

## 1. Introduction

Melting, a fundamental phase transition in condensed matter systems, is a complex process with far-reaching implications across various scientific disciplines and industrial applications. This transition involves the transformation of an ordered crystalline structure into a disordered liquid phase, characterized by significant changes in atomic mobility and structural properties. While macroscopic properties of melting are well-understood, the atomic-scale aspects of this phenomenon remain elusive. Traditionally, it was believed that melting preferentially initiates at crystal surfaces, grain boundaries, and dislocations [1]. However, recent research has revealed that it often begins at defect sites within crystal structures, challenging the conventional view [2]. The essence of melting lies in the dramatic increase in atomic mobility as a material approaches and undergoes the structural transition from a solid to a liquid. Therefore, the ability to visualize real-time structural changes at the relevant length scale is crucial for both underlying scientific principles and practical applications.

In recent years, advancements in experimental techniques, such as high-resolution electron diffraction and in-situ transmission electron microscopy (TEM), have enabled direct investigation of nanomaterial structures and dynamics under various conditions. For instance, the development of closed environmental cells has broadened the scope of electron microscopy, allowing for the study of materials that easily vaporize from solid specimens [3,4,5]. Modern encapsulation techniques, such as silicon nitride [6,7,8], graphene [9,10,11,12], and their hybrids [13,14,15], offer the necessary physical robustness and electron transparency. These features enable the safe and efficient handling of liquid-containing specimens within the extreme high vacuum conditions of electron optics. Liquid phase electron microscopy (LPEM) has been employed to explore a variety of chemicals, ranging from aqueous solutions [16,17,18] to organic solvents [19,20,21], and from acidic [22] to basic solutions [23]. This approach has proven effective in characterizing nanomaterial formation dynamics [9,16,24,25], electrochemical reactions [26,27,28], and in capturing high-resolution images of wet biomolecules [18,29] under specialized configurations. However, many in-situ reactions occurring within an environmental cell TEM can trigger undesirable side reactions, potentially altering the reaction mechanisms compared to ex-situ conditions. To better understand the mechanisms behind these redox-related etching, nucleation, and electrochemical reactions, radical chemistry is often considered [30,31,32], especially since the direct energy transfer from highly accelerated incident electron beams involves very small cross-sections. Consequently, minimizing the effects of reactive radicals should be a primary consideration unless they are intentionally used to initiate the reaction during in situ electron microscopy. Several methods are available to suppress or neutralize radical generation during electron microscopy, ensuring more accurate observations. For instance, cryogenic electron microscopy mitigates radiolytic decomposition by reducing the mobility and reaction rate of generated radicals [33]. Also, graphene-based liquid cell techniques exploit the radical-scavenging properties of graphene derivatives [11,34,35], and low-dose electron microscopy [18] minimizes the production of radical molecules.

In this work, we circumvented the effects of radiolysis by using a radically inert liquid and investigated the charge-induced melting of metal nanoparticles in TEM. In our finding, even at a high electron dose rate of 10^5^ e^−^/Å^2^·s, radiolysis-induced etching or precipitation was absent, with only charge-induced disordering of colloidal nanoparticles being observed. We examined the changes in morphology and crystallinity of nanoparticles dispersed in the liquid under substantial electron irradiation. The experimental configuration ensured that suspended nanoparticles did not contact the membrane during melting, allowing nucleation to occur independently of membrane interaction. This approach enabled probing the solid-liquid phase transition while preventing direct vaporization or sublimation into the vacuum. In-situ and postmortem analyses revealed that the amorphous and crystalline phases transition between each other during the melting process, closely mirroring the dense-phase formation stage in nucleation.

## 2. Experimental Methods

The Au nanoparticles utilized in our experiments were approximately 30 nm in diameter and sourced from certified reference materials (CRM) provided by the Korea Research Institute of Standards and Science (KRISS). For the in situ liquid-cell TEM experiments, we employed a Poseidon liquid holder from Protochips, Morrisville, NC, USA. The experiments were conducted using a liquid microelectromechanical system (MEMS) chip with a window size of 550 × 50 μm and a spacer thickness of 50 nm for electron irradiation. Additionally, we used the Poseidon electrochemistry E-chip for potential measurements. The Si_3_N_4_ membrane in the liquid cell was also approximately 50 nm thick, facilitating electron beam irradiation while preserving the liquid environment. The Au nanoparticles were suspended in solvents, including acetonitrile and water, and the suspension was carefully applied to the liquid MEMS chip. The static-type liquid phase TEM experiments were then performed, during which electron beam irradiation was applied, and potential measurements were recorded as part of the experimental procedure.

The real-time TEM images from the liquid cell were acquired using a FEI Tecnai F30 (Thermo Fisher, Waltham, MA, USA) equipped with a Gatan OneView camera at the Korea Research Institute of Standards and Science (KRISS). The microprobe mode was used with a fixed electron beam size of 50 nm and 190 nm, corresponding to 1.3 × 10^5^ e^−^/Å^2^·s and 9.2 × 10^3^ e^−^/Å^2^·s, respectively. The screen current for the electron beam was 4 nA. The TEM operated at 300 kV with an energy spread of approximately 1 eV (ΔE/E ~ 10^−6^), as determined by the zero-loss peak in electron energy loss spectroscopy.

## 3. Results and Discussion

### 3.1. Charge-Induced Breakdown of Nanoparticles in a Liquid Cell Electron Microscopy

Compared to water-filled liquid environmental cells, Au nanoparticle dispersions in acetonitrile exhibit greater tolerance to high electron beam doses due to reduced radical generation. In aqueous LPEM systems, primary decomposition and subsequent chain reactions produce reactive oxygen species (ROS) such as OH,·O_2_^−^,·O_2_^2−^, HO_2_, HO_3_,·O_3_, and other unstable radicals [30]. These radicals contribute to radiolytic decomposition by breaking material bonds during the propagation stage. While acetonitrile also undergoes bond dissociation under high energy intake, its primary decomposition products, ·H and CH_2_CN, are more stable than those from water. The bond dissociation energy (BDE), a measure of radical stabilization enthalpy (RSE), of the·CH_2_CN radical (401.71 kJ mol^−1^) is lower than that of the hydroxyl radical (497.1 kJ mol^−1^), indicating its greater stability. At steady state, stable methyl radicals (CH_3_, BDE = 439.3 kJ mol^−1^) and reactive cyanide radicals (CN, BDE = 527.6 kJ mol^−1^) are generated, but their saturation concentrations are approximately 10^5^ times lower than the primary species [36,37,38].

**Figure 1:**
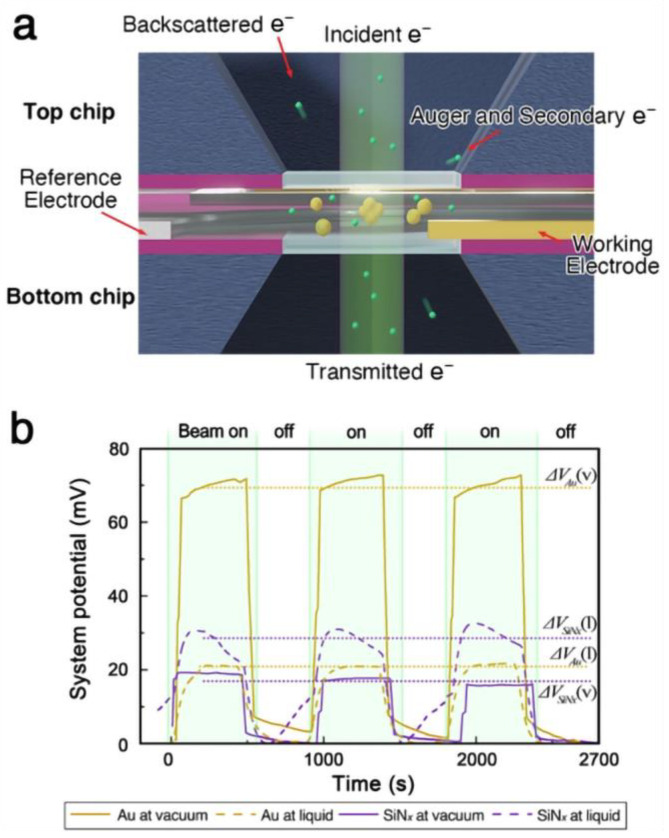
(**a**) Schematics of an electrically insulated liquid cell configuration with electrodes. (**b**) The potential between the working electrode and reference electrode was measured during electron illumination of the environmental cell.

While the use of radiolytic intervention-suppressed liquid cells allow for electron microscopy without undesirable side reactions at lower electron doses, higher irradiation levels introduce new challenges. The insulating properties of the liquid cell’s constituent elements, such as acetonitrile and silicon nitride [39], can lead to significant charge buildup during electron illumination. This charge accumulation, caused by the ejection of secondary and Auger electrons, can negatively impact imaging quality and even lead to sample decomposition at extreme levels [40,41,42,43]. Unlike conventional conducting carbon-coated grid specimens or graphene liquid cells, the insulating nature of acetonitrile and silicon nitride hinders charge dissipation, exacerbating the issue. The internal potential, induced by electron illumination, could potentially be measured using off-axis holography [44,45]. However, this technique is challenging to implement in liquid cell microscopy due to the lack of a suitable vacuum phase reference. As an alternative, the relative electrical potential of the liquid holder system was measured directly using an electrically isolated environmental cell setup (Figure 1a). To ensure accurate potential measurement, the dielectric materials and electron-transparent window were replaced with a 300 nm thick membrane, deviating from the conventionally used 50 nm-thick liquid MEMS chip.

When the liquid cell was filled with acetonitrile, the magnitude of potential change decreased compared to the vacuum case (Figure 1b). This reduction is expected, as the effective escape surface for low-energy secondary electrons remains constant while the illuminated volume increases due to the liquid. Although the potential change in the liquid cell system (20 ± 0.3 mV) was smaller than that observed for gold in vacuum (70.4 ± 1.7 mV), it was comparable to the potential measured during the illumination of an insulator in vacuum (17.4 ± 1.3 mV). While the overall potential measurement of the liquid cell does not provide an accurate estimate of the gold nanoparticle’s charge state, it suggests that the illuminated area is positively charged relative to the surrounding system. Given that the cumulative charge in the illuminated liquid column is similar to that of the irradiated nitride membrane in vacuum, the material within the liquid cell is also likely to experience charge-induced structural changes under focused electron beam irradiation.

### 3.2. Morphology Change of Nanoparticle During Charge-Induced Melt

**Figure 2:**
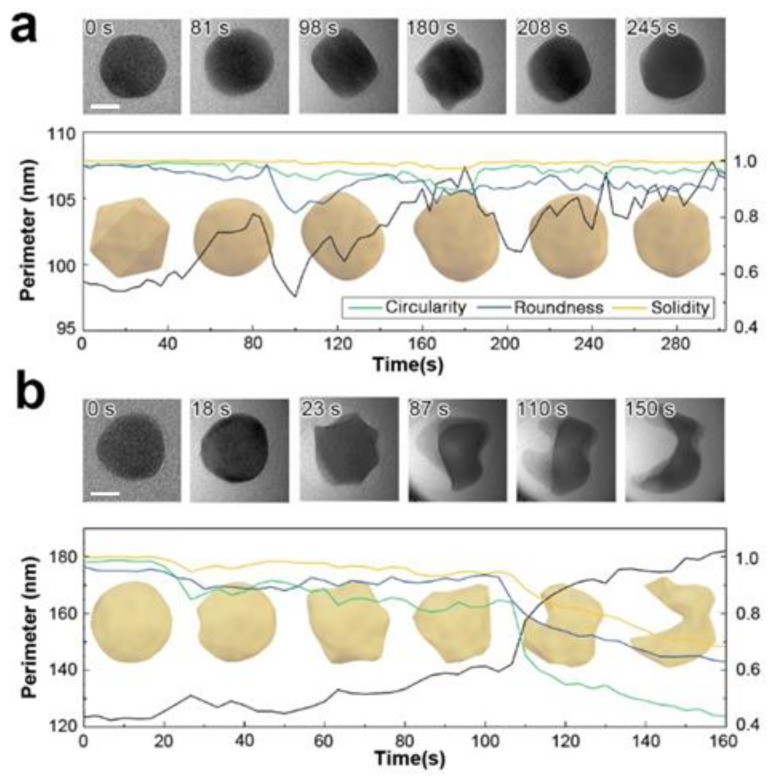
Morphological evolution of Au nanoparticles dispersed in acetonitrile under varying electron doses. (**a**) At a moderate electron dose rate of 9.2 × 10^3^ e^−^/Å^2^·s, typically used during liquid phase electron microscopy (LPEM), the nanoparticle exhibits slight surface deformation and viscous behavior. (**b**) At a high electron dose rate of 1.3 × 10^5^ e^−^/Å^2^·s, comparable to high-resolution TEM conditions for solid phases, the nanoparticle undergoes significant deformation, displaying behavior characteristic of solid-liquid phase transformation. Scale bar is 10 nm.

Focused electron beam irradiation can induce ballistic damage in only 16% of atoms with a single positive charge, resulting from broken bonds [46]. In gold nanoparticles, this damage can manifest as surface fluctuations, atomic relocation, loss of crystal ordering, or field evaporation [47]. However, in an acetonitrile liquid cell, evaporation is prevented by the surrounding liquid, leading to local mass effusion. To investigate the effects of charge on particle deformation in a liquid, a gold nanoparticle suspended in an acetonitrile solution was irradiated with an electron probe current of 4 nA and a diameter of 185 nm (9.2 × 10^3^ e^−^/Å^2^·s). A single floating Au nanoparticle, initially icosahedral, underwent a shape transformation, initially becoming spherical and then elongating in one direction. The elongated cuboid exhibited a minimal roundness factor and a maximum aspect ratio. A twin boundary formed along the major axis, confirming the particle’s single-crystal structure. Upon further irradiation to 1.7 × 10^6^ e^−^/Å^2^ (180 s), the particle surface became viscous and behaved like a dense liquid, with a minimum circularity factor indicating an elongated perimeter and complex surface morphology. While additional irradiation did not alter the particle’s overall shape, the perimeter remained larger than its original state (Figure 2a). We note that, at this electron dose, aqueous liquid cells would typically undergo radiolytic decomposition and particle explosion, but the nanoparticle in this case maintained its original projection area and did not experience radiolytic reactions.

At a significantly higher dose of 1.3 × 10^5^ e^−^/Å^2^·s, dramatic deformation of the particle is observed (Figure 2b). Liquefaction of the gold particle initiates at 2.3 × 10^6^ e^−^/Å^2^ (18 s) when small protrusions appear on the surface. Up to this point, the particle maintains its original crystallinity, as evidenced by the observable diffraction contrast throughout its structure. The particle retains its solid state until 100 s of irradiation, beyond which it loses convexity and structural integrity. When an equivalent electron dose is applied to a multi-particle system, similar structural disordering occurs following particle coalescence. Particle fusion also occurs slowly in vacuum under similarly focused irradiation. On rigid supports, particles must overcome the anchoring force towards the membrane to form oriented attachments. In contrast, free-floating nanoparticles encounter lower resistance to movement. This reduced resistance facilitates easier steric relocation, promoting attachment and mass transport throughout the joined region.

### 3.3. Crystallographic Evolution of Nanoparticle During Charge-Induced Melt

**Figure 3:**
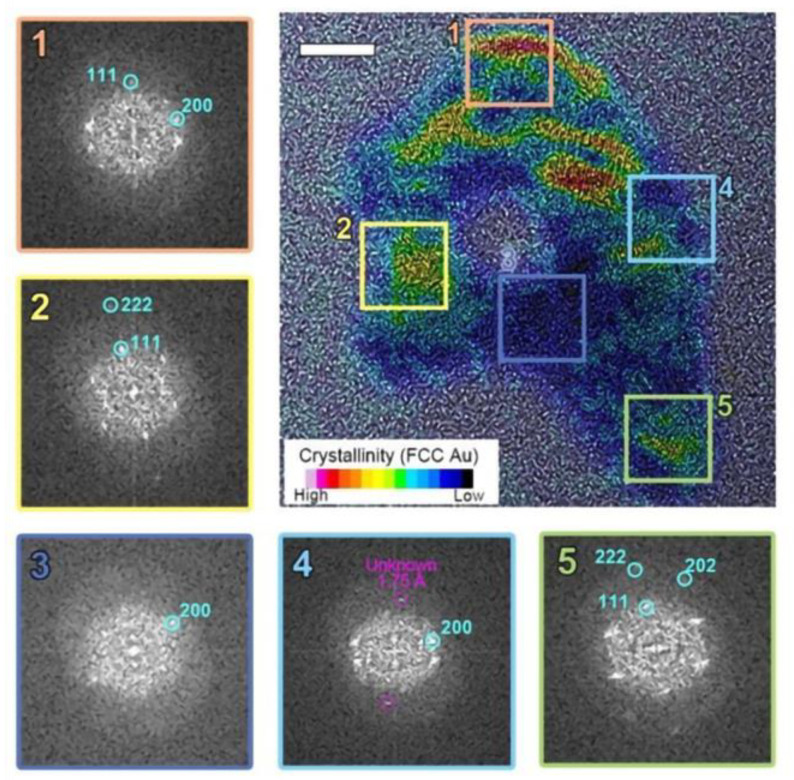
The postmortem crystallinity mapping of the particle revealed a combination of crystalline FCC Au and amorphous Au within a single particle. Regions 1, 2, and 5 show a well-ordered face-centered cubic (FCC) structure, while regions 3 and 4 exhibit amorphous phases.

Unlike radiolytic decomposition reactions, we found no chemical changes during this structural deformation process. The particle’s redox state remains unaltered, and no metastable reactants form. Consequently, studying the crystallographic evolution through atomic movement during this charge-induced melting provides valuable insights into structural changes during the solid-liquid phase transition. Several crystallinity changes, including diffraction fringes and twin boundaries, were observed during the in situ melting of Au nanoparticles. However, detailed atomic movements could not be rendered in real-time due to the spatial resolution limitations of liquid-phase electron microscopy (LPEM). To overcome this constraint and acquire high-quality crystallographic information, postmortem analysis of dissociated particles was conducted (See Figure 3).

Typical Au nanoparticles consist of multiple crystallographic grains with a face-centered cubic (FCC) structure. Initially, these grains appear randomly oriented, forming a quasi-spherical shape as shown in Figure 2. However, after electron beam irradiation, the particle morphology begins to deform. The elongated particle develops internal voids and locally amorphous regions during the liquid state. Selective inverse Fourier transform imaging (Figure 3) reveals a mixture of crystalline and amorphous regions throughout the particle, with non-identifiable metastable lattice planes (d = 1.75 Å) distributed within the amorphous region. As shown in Figure 3, two-dimensional FFT analysis on the selected regions of interest in the TEM image reveals that the crystalline phase within a single melted region tends to adopt a preferred orientation, even when separated by amorphous and void regions. Here, we note that the permanent damage to the sample due to heating within the irradiated volume is negligible, as the expected temperature of the gold nanoparticles is far below their melting point. Furthermore, radiolysis is likely suppressed at the electron beam dose rate employed in this experiment due to the nature of the solvent used and the absence of significant reactive species. Therefore, we attribute the rich morphological changes and phase transitions observed in this work primarily to the charging effect.

To further scrutinize our observations, we examined the changes in particle shape as it is continuously exposed to electron beam irradiation. As shown in Figure 2a, Au nanoparticles initially consist of polycrystalline FCC structures. Upon electron beam irradiation, the particle perimeter becomes smoother and eventually exhibits signs of deformation. The particle edges also show a loss of crystalline structure in localized areas. We attribute this effect to the formation of highly defective gold layers near the surface. The highly energized Au atoms near the surface with increased atomic mobility lead to surface deformation until a new crystal equilibrium is found (See Figure 2a). This may suggest partial surface melting of the particle, where surface atoms are considerably more mobile than those in the interior. In contrast, in the higher fluence case (Figure 2b), surface deformation continues to develop to likely an amorphous phase, which is accompanied by a significant loss of material. Notably, the particle did not recover its original FCC structure but retained a highly distorted shape. This observation implies that the particle has melted. In some particles, a perfectly ordered single crystalline FCC structure is observed, even in remote discrete areas within the same particle (regions 2 and 5 in Figure 3). This may suggests that the formation of voids and locally amorphous regions occurs after at least one crystalline arrangement during the liquefaction of the solid particle. In the melting process, amorphization and crystallization could both be present, with each phase competing due to local fluctuations and instabilities that are observed during the nucleation process [24,48]. To accurately capture and understand these intricate transient processes, high-speed, highly sensitive detectors operating at sub-millisecond timescales are essential [49]. However, the development and implementation of such advanced technology fall outside the scope of this study.

Regarding the effect of nanoparticle size on charge-induced melting, it is established that size plays a crucial role in determining the melting behavior of nanoparticles [50]. Smaller nanoparticles generally exhibit lower melting points than larger ones due to their higher surface-to-volume ratios. This size effect results in more pronounced transformations under the same conditions, with smaller particles melting or deforming at lower electron doses or temperatures. In the case of charge-induced melting, the size of the nanoparticle directly influences the extent of charge accumulation and the resulting morphological changes. Due to their larger relative surface area, smaller particles tend to accumulate more charge per unit volume, which can intensify the melting effect. As a result, smaller particles may experience greater structural instability under comparable electron beam irradiation, leading to faster deformation and melting.

Although we did not specifically investigate size-dependent melting in the current study, it is reasonable to expect that nanoparticle size would significantly influence melting behavior. Based on theoretical models and prior research, smaller nanoparticles are likely to melt at lower thresholds compared to larger particles under similar charge conditions. Conversely, larger nanoparticles may require higher electron doses or prolonged exposure times to exhibit comparable melting dynamics. In our study, where we examined a 30-nm diameter particle, we observed a distinct charge-induced melting phenomenon at a specific threshold dose. We anticipate that future experiments exploring a range of particle sizes will further clarify and validate the relationship between nanoparticle size and charge-induced melting behavior.

## 4. Conclusions

We investigate the nanoscale dynamics of melting in metal nanoparticles by circumventing radiolysis challenges in liquid-phase electron microscopy. Using a radically inert liquid (acetonitrile) and high-dose electron irradiation, we observed charge-induced melting of gold nanoparticles without radiolytic interference. Our findings imply an elaborate melting process characterized by electron beam fluence dependent transitions between amorphous and crystalline phases, involving multiple stages. The observation of preferred crystalline orientations within melted regions suggests that void and locally amorphous area formation occurs after at least one crystalline arrangement during liquefaction. These observations challenge conventional understanding of melting and provide new perspectives on the structural evolution of materials during phase transitions. The methodology demonstrated in this work opens new avenues for investigating other critical phase transitions and may have implications for various fields, including materials science, nanotechnology, and crystal growth studies.

## Data Availability

The data presented in this study are available on request from the corresponding author.
